# Supplementary figures and images for: Phosphazene-Containing Epoxy Resins Based on Bisphenol F with Enhanced Heat Resistance and Mechanical Properties: Synthesis and Properties
Source: Polymers (Basel). 2022 Oct 27;14(21):4547. doi: 10.3390/polym14214547 (PMC9655627; doi:10.3390/polym14214547)

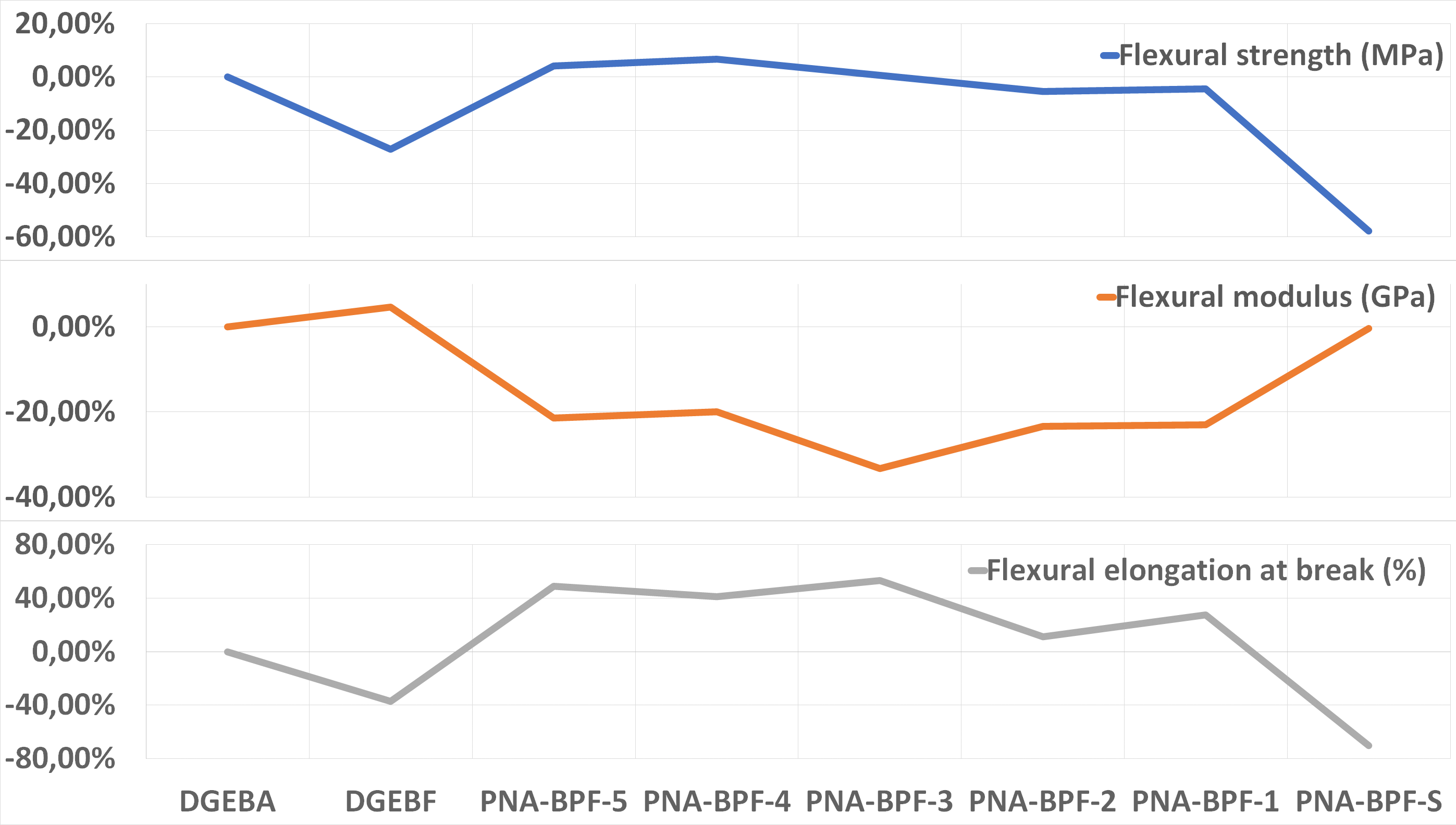

Supplement: Supplementary file 1 [file polymers-14-04547-s001.zip › Figure S1. Flexural properties.png]

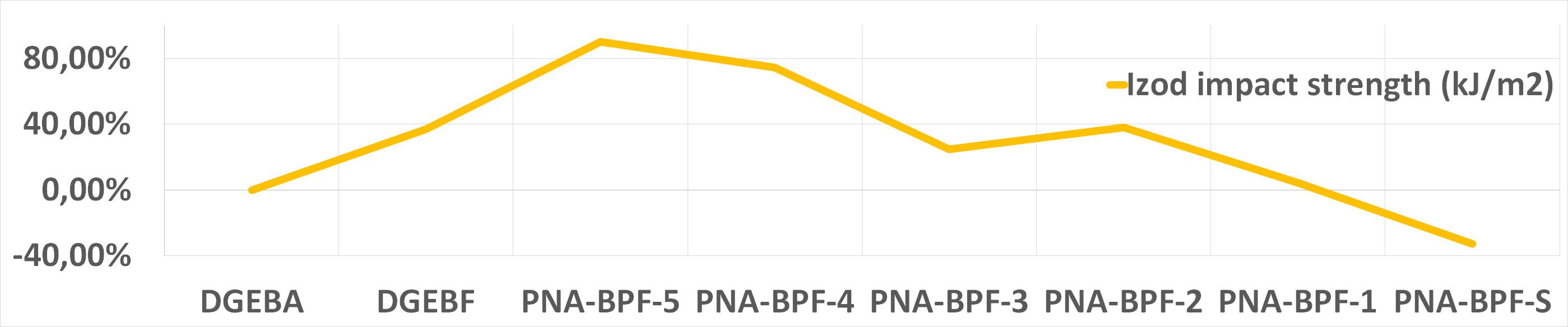

Supplement: Supplementary file 1 [file polymers-14-04547-s001.zip › Figure S2. Impact strength.png]

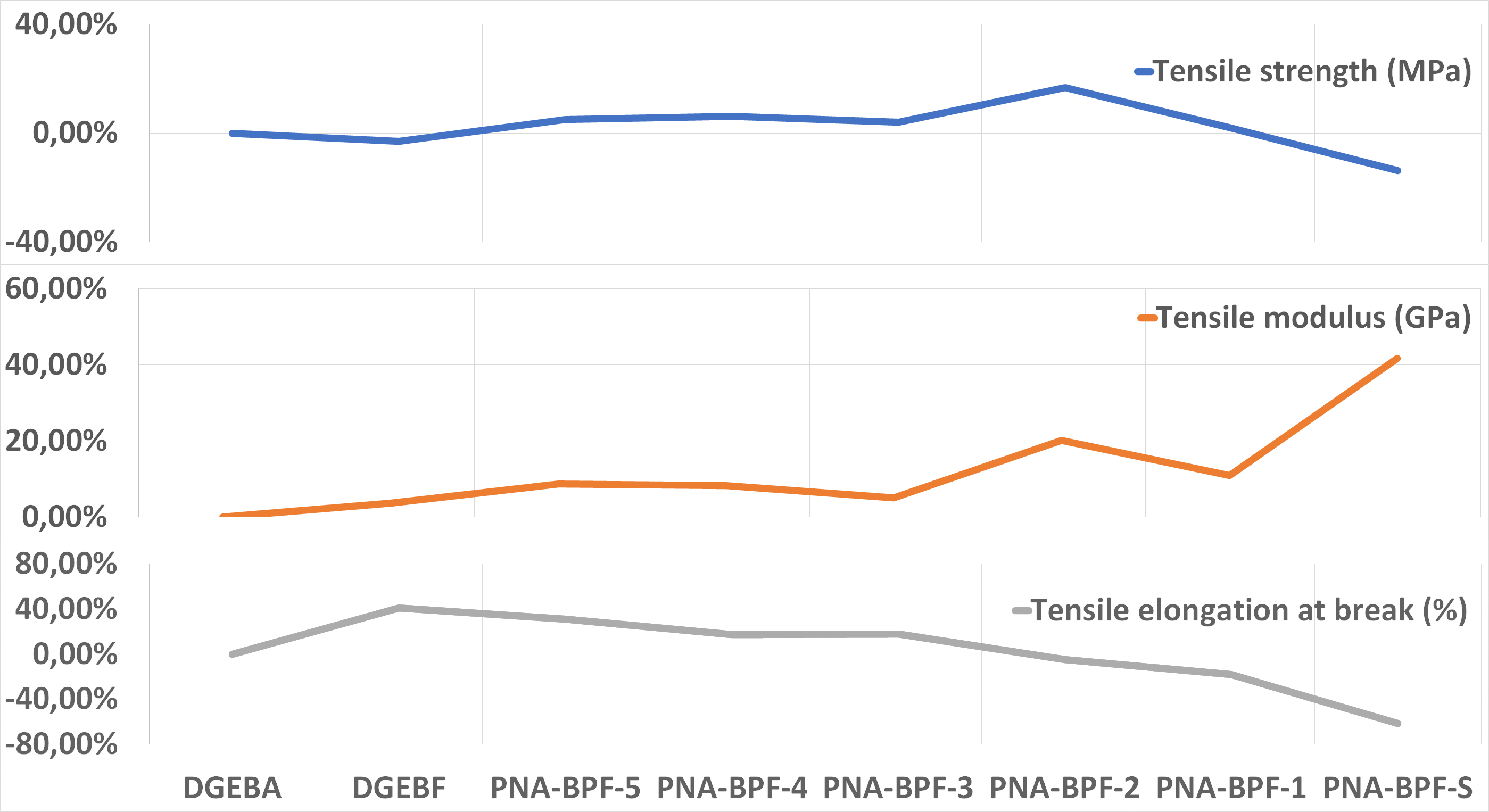

Supplement: Supplementary file 1 [file polymers-14-04547-s001.zip › Figure S3. Tensile properties.png]
